# Genetic parameters for uniformity of harvest weight and body size traits in the GIFT strain of Nile tilapia

**DOI:** 10.1186/s12711-016-0218-9

**Published:** 2016-06-10

**Authors:** Jovana Marjanovic, Han A. Mulder, Hooi L. Khaw, Piter Bijma

**Affiliations:** Animal Breeding and Genomics Centre, Wageningen University and Research, PO Box 338, 6700 AH Wageningen, The Netherlands; Department of Animal Breeding and Genetics, Swedish University of Agricultural Sciences, Box 7023, 75007 Uppsala, Sweden; WorldFish, Jalan Batu Maung, 11960 Bayan Lepas, Penang Malaysia

## Abstract

**Background:**

Animal breeding programs have been very successful in improving the mean levels of traits through selection. However, in recent decades, reducing the variability of trait levels between individuals has become a highly desirable objective. Reaching this objective through genetic selection requires that there is genetic variation in the variability of trait levels, a phenomenon known as genetic heterogeneity of environmental (residual) variance. The aim of our study was to investigate the potential for genetic improvement of uniformity of harvest weight and body size traits (length, depth, and width) in the genetically improved farmed tilapia (GIFT) strain. In order to quantify the genetic variation in uniformity of traits and estimate the genetic correlations between level and variance of the traits, double hierarchical generalized linear models were applied to individual trait values.

**Results:**

Our results showed substantial genetic variation in uniformity of all analyzed traits, with genetic coefficients of variation for residual variance ranging from 39 to 58 %. Genetic correlation between trait level and variance was strongly positive for harvest weight (0.60 ± 0.09), moderate and positive for body depth (0.37 ± 0.13), but not significantly different from 0 for body length and width.

**Conclusions:**

Our results on the genetic variation in uniformity of harvest weight and body size traits show good prospects for the genetic improvement of uniformity in the GIFT strain. A high and positive genetic correlation was estimated between level and variance of harvest weight, which suggests that selection for heavier fish will also result in more variation in harvest weight. Simultaneous improvement of harvest weight and its uniformity will thus require index selection.

**Electronic supplementary material:**

The online version of this article (doi:10.1186/s12711-016-0218-9) contains supplementary material, which is available to authorized users.

## Background

In animal breeding, particular attention is paid to improving the mean level of traits through selection and this has been successful for many breeding programs. One such successful example is the genetically improved farmed tilapia (GIFT) project, which was led at WorldFish [1] and resulted in a line of tilapia known as the GIFT-strain. For this strain, a substantial realized genetic gain (>100 %) was achieved through 12 generations of genetic improvement for body weight at harvest [2, 3]. However, it is often desirable not only to improve the level of a trait, but also to reduce its variability [4, 5], because significant variation around the optimal value of a trait can have a negative impact on production performance, both in livestock and aquaculture [5–7]. In fish farming, differences in size among individuals are generally associated with competition for food within a group and the resulting feeding hierarchy [6, 8, 9]. The phenotypic coefficient of variation (CV) for body weight, apart from indicating variation of the trait is also an indicator of competitive interactions within a population [8]. For the GIFT strain, the CV ranges from 40 to 60 %, which is considered a high value [10].

Although good management during the grow-out phase can help reduce the CV, as noted by Ponzoni et al. [2], its average value across eight generations of GIFT remained at around 40 %. A common approach in fish farming to decrease phenotypic variation in body size and weight is to grade or sort fish into groups, according to size. If fish are not graded, the large variation in weight and size at harvest reduces their market value and has animal welfare consequences [11, 12]. From the point of view of fish farmers, uniformity of growth and body size is one of the key traits to be improved [11]. From the consumer’s point of view, weight but also body size and appearance traits, play an important role in buying decisions [13–15].

An alternative approach to management procedures for reducing the variability of a trait is selective breeding. Selection for more uniform individuals requires that the variability of the trait itself has a genetic component i.e. that there is genetic variation, which is also known as genetic heterogeneity of environmental (residual) variance [16, 17]. In this case, within a population, some animals will be less prone than others to phenotypic changes in response to small environmental fluctuations, and thus will have a more stable performance. Several studies on livestock and laboratory animals have demonstrated the existence of genetic differences in residual variance among genotypes and have quantified their magnitude [7, 16, 18–28]. In aquaculture species, evidence for substantial genetic heterogeneity of residual variance comes from three studies on body weight in salmonids [29–31]. A previous study on uniformity in Nile tilapia that analyzed the standard deviation of harvest weight using a traditional linear mixed model indicated a genetic basis for variability of harvest weight [12]. However, to date, variability of harvest weight in Nile tilapia has not been analyzed at the variance level using double hierarchical generalized linear models (DHGLM). The DHGLM is a novel approach that can be used to study uniformity of individual trait values. The advantage of DHGLM compared to analyzing variance or the standard deviation of a group is that it can take into account systematic effects on the variance of the individual record level such as sex of the fish. The genetic basis of the variability of body size traits has not been explored in any species, except in humans for height [32].

The main objective of our study was to investigate the potential for genetic improvement of uniformity of harvest weight and body size traits in the GIFT strain. For this purpose, we analyzed within-family variance of harvest weight, body length, depth, and width, by applying a DHGLM to individual trait values [33]. To quantify the genetic relationship between the level and the variance of these traits, we also estimated the genetic correlation between these two components.

## Methods

### Environment

We used data that were obtained from an experiment that was specifically designed to estimate indirect genetic effects (IGE) for growth rate in the GIFT strain [34]. This experiment was carried out between 2009 and 2012 at the Jitra Aquaculture Extension Centre of the Department of Fisheries, which is managed by WorldFish and located at Kedah State of Malaysia. WorldFish complies with the Malaysian laws on animal experiments. During this experiment, four batches of fish were produced, i.e. one batch each year (batch named per year). However, for the last batch (2012), a high level of mortality occurred due to extreme weather conditions, which resulted in an insufficient number of records, and thus it was excluded from the analysis.

### Experimental design

**Fig. 1:**
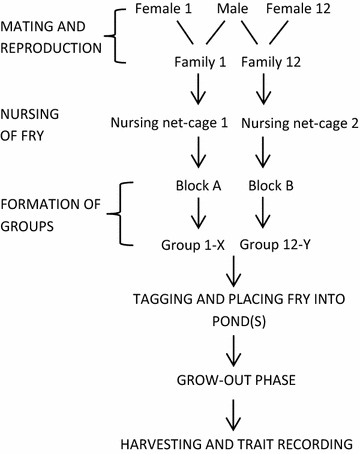
Outline of the experimental design for two paternal families. X represents any family from Block A, other than family 1; Y represents any family from Block B, other than family 12; an example of Block A is in Figure S1 (see Additional file 1: Figure S1)

To produce families, the GIFT breeding program uses a nested-mating design, where one male is mated to two females. For this work, we used the same mating scheme to produce the experimental fish, and thus two full-sib families were obtained from each father. Each full-sib family contributed 80 offspring to the experiment. Fry that belonged to the same full-sib family were nursed together and separately from other families. During the grow-out phase, fish were kept in groups. Before placing each fish in a group, they were individually identified with a PIT (Passive Integrated Transporter) tag. Following the optimal design for the estimation of IGE [35], families were assigned to groups so that each group consisted of members of two distinct, unrelated families. Both families contributed eight randomly selected individuals to each group to form groups of 16 members. Therefore, each family of 80 offspring contributed to 10 distinct groups (i.e. 80/10 members per group). Unique combinations of families in groups were created using a block design, with 11 families per block, where each family was combined only once with the other ten families in the same block. Hence, there were 55 family combinations i.e. groups, per block. Figure S1 (see Additional file 1: Figure S1) shows an example of the block design. If the number of available families for the last block was less than 11, an incomplete block was used with all the remaining families. An outline of the various steps that were carried out for each batch is in Fig. 1.

The groups were kept in net-cages that were placed in earthen ponds in rows and columns. For each batch, two ponds were available. Due to the small number of fish available for batch 2010, only one pond was used. The groups for each block were distributed randomly and as evenly as possible over both ponds. Thus, the 55 groups of a block were split into 27 groups for pond 1, and 28 groups for pond 2.

During the grow-out phase, fish were fed with commercial dry pellets containing 32 % of protein; the amount of pellets (3 to 5 % of average live weight) and feeding frequency (twice a day) were the same as for the GIFT selective breeding population. However, because the fish were kept in net-cages rather than in communal rearing, the feeding strategy differed from that in the standard GIFT program. Rather than spreading the food over the entire surface of a pond, it was placed in the corner of each net-cage so that the fish could express their competitive tendency (see Discussion). More details on the experiment are in Khaw et al. [12, 34]. The GIFT technology manual provides a description of key husbandry procedures [36].

### Records

Fish were harvested 5 to 8 months after the grow-out period, when the average weight ranged from 200 to 250 g. At harvest, the following traits and parameters were recorded: live body weight (g), body measurements (length, depth, and width, in cm), tag number, sex, pond, and net-cage label. The age at harvest of each fish was computed from the recorded spawning and harvesting dates [34]. Over three batches, phenotypic observations on body weight and body measurements at harvest were available for 6330 fish from 493 groups.

Ideally, each group should contain 16 individuals at harvest. However, due to mortality, some groups contained very few individuals, and a threshold was set for group and family size. Thus, groups that contained less than seven individuals in total or less than three fish per family were discarded, which reduced the number of groups to 446. With two families in each group, 892 family-by-group combinations and 6090 individual records were available for each trait. Table 1 shows the number of observations at harvest (full dataset) and number of observations used in the analysis (edited or reduced dataset). The pedigree consisted of 34,517 records that traced the GIFT population back seven generations.

**Table 1:** Number of groups, families per group, and individuals at harvest (C-complete dataset) and after editing (R-reduced dataset)

Batch	Families		Groups		Families per group		Individuals	
	C	R	C	R	C	R	C	R
2009	66	66	209	188	418	376	2565	2461
2010	33	31	45	37	90	74	509	464
2011	68	68	239	221	478	442	3256	3165
Total	167	165	493	446	986	892	6330	6090

### Statistical analysis

The environmental component in the phenotypic variation of a trait can be measured either on the same individual for which repeated observations are available or on the individuals belonging to the same family [37]. In our dataset, body weight and body measurements were recorded at harvest. Hence, only one record for each trait was available for each individual, but eight observations were recorded per family per group. To analyze the genetic heterogeneity of the environmental variance, different approaches have been proposed [37] and we chose a DHGLM that models the residual variance of individual observations on the exponential scale, and can be interpreted as a multiplicative model [17]. On the level of the natural logarithm, the multiplicative model becomes additive.

Sire and dam, group, kin, and social maternal effect were included as random effects. A group effect was included to account for non-heritable indirect effects, which create a non-genetic covariance among individuals within the same group [38]. If this covariance is present but not accounted for, it can cause bias in the estimated genetic parameters [39]. According to the kin selection theory, relatives can cooperate with each other [40, 41], thus a non-genetic covariance between group mates belonging to the same family can arise. Therefore, we included a kin effect to account for this source of non-genetic covariance i.e. between group mates of the same family compared to group mates of the other family within a group [34]. Finally, a social maternal effect was included that accounts for the non-genetic effect of the common maternal environment of one full-sib family on the performance of the other full-sib family in the group [12]. In other words, we fitted a non-genetic effect of the mother of a full-sib family on the trait values of the other full sib family kept in the same group. Hence, we termed this effect “social”, because it is expressed in the trait values of the social partners of the offspring of a mother, rather than in the offspring themselves.

### Double hierarchical generalized linear models (DHGLM)

Lee and Nelder [42] developed a framework for the DHGLM, where level and residual variance of a trait can be modeled jointly with specified random effects. This approach has been applied in animal breeding by Rönnegård et al. [33] who implemented the DHGLM in the statistical software SAS and ASReml 2.0 [33]. The DHGLM algorithm iterates between two sets of mixed model equations i.e. a linear mixed model for the phenotypic records and a generalized linear mixed model for the response variable $\phi_{i}$. $\phi_{i}$ is defined as $\phi_{i} = E\left( {\hat{e}_{i}^{2} /\left( {1 - h_{i} } \right)} \right)$, where $\hat{e}_{i}^{2}$ is the squared residual for the $i{\text{th}}$ observation and $h_{i}$ is the diagonal element of the hat matrix of ${\mathbf{y}}$, corresponding to the same individual [33, 43]. As $\phi$ follows a $\chi^{2}$ distribution, $\hat{e}_{i}^{2} /\left( {1 - h_{i} } \right)$ can be linearized using a log link function so that ${ \log }\left( \phi \right) = { \log }\left[ {\hat{e}_{i}^{2} /\left( {1 - h_{i} } \right)} \right]$ [33]. Instead of using a log link function, ${ \log }\left[ {\hat{e}_{i}^{2} /\left( {1 - h_{i} } \right)} \right]$ can be linearized using a first order Taylor-series expansion as shown by Felleki et al. [44], which results in the response variable $\psi_{i} = { \log }\left( {\hat{\upsigma }_{{e_{i} }}^{2} } \right) + \left( {\left\{ {\left[ {e_{i}^{2} /\left( {1 - h_{i} } \right)} \right] - \left. {\hat{\upsigma }_{{e_{i} }}^{2} } \right\}} \right./\hat{\upsigma }_{{e_{i} }}^{2} } \right)$, where $\hat{\upsigma }_{{e_{i} }}^{2}$ denotes the predicted residual variance for observation $i$, and *e*_*i*_ is the residual for individual *i*. Due to linearization, a bivariate DHGLM can then be used:

$$\begin{aligned} \left[ {\begin{array}{*{20}c} {\mathbf{y}} \\ {\varvec{\uppsi}} \\ \end{array} } \right] & = \left[ {\begin{array}{*{20}c} {\mathbf{X}} & \mathbf 0 \\ \mathbf 0 &\quad {{\mathbf{X}}_{{\text{V}}} } \\ \end{array} } \right]\left[ {\begin{array}{*{20}c} {\mathbf{b}} \\ {{\mathbf{b}}_{{\text{v}}} } \\ \end{array} } \right] + \left[ {\begin{array}{*{20}c} {{\mathbf{Z}}_{{{\text{Par}}}} } & \mathbf 0 \\ \mathbf 0 &\quad {{\mathbf{Z}}_{{{\text{Par}}_{{\text{v}}} }} } \\ \end{array} } \right]\left[ {\begin{array}{*{20}c} {\mathbf{a}} \\ {{\mathbf{a}}_{{\text{v}}} } \\ \end{array} } \right] \\ & \quad + \left[ {\begin{array}{*{20}c} {\mathbf{V}} &\mathbf 0 \\ \mathbf 0 &\quad {{\mathbf{V}}_{{\text{v}}} } \\ \end{array} } \right]\left[ {\begin{array}{*{20}c} {\mathbf{g}} \\ {{\mathbf{g}}_{{\text{v}}} } \\ \end{array} } \right] + \left[ {\begin{array}{*{20}c} {\mathbf{S}} & \mathbf 0 \\ \mathbf 0 & \quad {{\mathbf{S}}_{{\text{v}}} } \\ \end{array} } \right]\left[ {\begin{array}{*{20}c} {\mathbf{k}} \\ {{\mathbf{k}}_{{\text{v}}} } \\ \end{array} } \right] \\ &\quad + \left[ {\begin{array}{*{20}c} {\mathbf{U}} & \mathbf 0 \\ \mathbf 0 &\quad {{\mathbf{U}}_{{\text{v}}} } \\ \end{array} } \right]\left[ {\begin{array}{*{20}c} {\mathbf{m}} \\ {{\mathbf{m}}_{{\text{v}}} } \\ \end{array} } \right] + \left[ {\begin{array}{*{20}c} {\mathbf{e}} \\ {{\mathbf{e}}_{{\text{v}}} } \\ \end{array} } \right], \\ \end{aligned}$$

where ${\mathbf{y}}$ is the vector of individual trait records (harvest weight, body length, depth, and width) and ${\varvec{\uppsi}}$ is the vector of response variables for the variance part of the model, expressed per individual ($\psi_{i}$ as defined above). ${\mathbf{b}}$ and ${\mathbf{b}}_{\text{v}}$ are the vectors of fixed effects, while ${\mathbf{a}}$ and ${\mathbf{a}}_{\text{v}}$ are the vectors of additive genetic effects of the sire and dam of each individual, with

$$\left( {\begin{array}{*{20}c} {\mathbf{a}} \\ {{\mathbf{a}}_{\text{v}} } \\ \end{array} } \right)\sim N\left( {\mathbf 0,\left[ {\begin{array}{*{20}c} {\upsigma _{\text{a}}^{2} } & {\upsigma _{{{\text{a}},{\text{a}}_{\text{v}} }} } \\ {\upsigma _{{{\text{a}},{\text{a}}_{\text{v}} }} } & {\upsigma _{{{\text{a}}_{\text{v}} }}^{2} } \\ \end{array} } \right] \otimes {\mathbf{A}}} \right),$$

where sire and dam variances are equal to a quarter of the additive genetic variance: $\upsigma _{{{\text{a}}_{{\left( {\text{v}} \right)}} }}^{2} = \frac{1}{4}\upsigma _{{{\text{A}}_{{\left( {\text{v}} \right)}} }}^{2}$, $\upsigma _{{{\text{A}}_{{\left( {\text{v}} \right)}} }}^{2}$ denoting the ordinary additive genetic variance. Note that we assume equal additive genetic variances for the sire and dam, i.e. $\upsigma _{{{\text{sire}}_{{\left( {\text{v}} \right)}} }}^{2} = \upsigma_{{{\text{dam}}_{{\left( {\text{v}} \right)}} }}^{2} = \upsigma _{{{\text{a}}_{{\left( {\text{v}} \right)}} }}^{2}$. ${\mathbf{g}}$ and ${\mathbf{g}}_{\text{v}}$ are the vectors of random group effects, with $$\left( {\begin{array}{*{20}c} {\mathbf{g}} \\ {{\mathbf{g}}_{\text{v}} } \\ \end{array} } \right)\sim N\left( {\mathbf 0,\left[ {\begin{array}{*{20}c} {\upsigma _{\text{g}}^{2} } & {\upsigma _{{{\text{g}},{\text{g}}_{\text{v}} }} } \\ {\upsigma _{{{\text{g}},{\text{g}}_{\text{v}} }} } & {\upsigma _{{{\text{g}}_{\text{v}} }}^{2} } \\ \end{array} } \right] \otimes {\mathbf{I}}} \right)$$; ${\mathbf{k}}$ and ${\mathbf{k}}_{\text{v}}$ are the vectors of random kin effects, with $$\left( {\begin{array}{*{20}c} {\mathbf{k}} \\ {{\mathbf{k}}_{\text{v}} } \\ \end{array} } \right)\sim N\left( {\mathbf 0,\left[ {\begin{array}{*{20}c} {\upsigma _{\text{k}}^{2} } & {\upsigma _{{{\text{k}},{\text{k}}_{\text{v}} }} } \\ {\upsigma _{{{\text{k}},{\text{k}}_{\text{v}} }} } & {\upsigma _{{{\text{k}}_{\text{v}} }}^{2} } \\ \end{array} } \right] \otimes {\mathbf{I}}} \right)$$; ${\mathbf{m}}$ and ${\mathbf{m}}_{\text{v}}$ are the vectors of social maternal effects, with $$\left( {\begin{array}{*{20}c} {\mathbf{m}} \\ {{\mathbf{m}}_{\text{v}} } \\ \end{array} } \right)\sim N\left( {\mathbf 0,\left[ {\begin{array}{*{20}c} {\upsigma _{\text{m}}^{2} } & {\upsigma_{{{\text{m}},{\text{m}}_{\text{v}} }} } \\ {\upsigma _{{{\text{m}},{\text{m}}_{\text{v}} }} } & {\upsigma _{{{\text{m}}_{\text{v}} }}^{2} } \\ \end{array} } \right] \otimes {\mathbf{I}}} \right)$$; and ${\mathbf{e}}$ and ${\mathbf{e}}_{\text{v}}$ are the vectors of random residuals that are assumed to be independent and normally distributed $$\left( {\begin{array}{*{20}c} {\mathbf{e}} \\ {{\mathbf{e}}_{\text{v}} } \\ \end{array} } \right)\sim N\left( {\mathbf 0,\left[ {\begin{array}{*{20}c} {{\mathbf{W}}^{ - 1} \upsigma _{\text{e}}^{2} } & 0 \\ 0 & {{\mathbf{W}}_{\text{v}}^{ - 1} \upsigma _{{{\text{e}}_{\text{v}} }}^{2} } \\ \end{array} } \right] \otimes {\mathbf{I}}} \right)$$ with scaling variances $\upsigma _{\text{e}}^{2}$ and $\upsigma _{{{\text{e}}_{\text{v}} }}^{2}$. The expectations for the scaling variances $\upsigma _{\text{e}}^{2}$ and $\upsigma _{{{\text{e}}_{\text{v}} }}^{2}$ are equal to 1, because ${\mathbf{W}}$ and ${\mathbf{W}}_{\text{v}}$ already contain the reciprocals of the estimated residual variances per record. The ${\mathbf{X}}\left( {{\mathbf{X}}_{\text{v}} } \right)$, ${\mathbf{Z}}\left( {{\mathbf{Z}}_{\text{v}} } \right)$, ${\mathbf{V}}\left( {{\mathbf{V}}_{\text{v}} } \right)$, ${\mathbf{S}}\left( {{\mathbf{S}}_{\text{v}} } \right)$ and ${\mathbf{U}}\left( {{\mathbf{U}}_{\text{v}} } \right)$ are known design matrices assigning observations to the level of fixed, sire and dam, group, kin, and social maternal effects for ${\mathbf{y}}\left( {\varvec{\uppsi}} \right)$, respectively. The weights, ${\mathbf{W}} = {\text{diag}}\left( {{\hat{\mathbf{\psi }}}} \right)^{ - 1}$ and ${\mathbf{W}}_{\text{v}} = {\text{diag}}\left( {\left( {1 - h} \right)/2} \right)$, are, together with vector ${\varvec{\uppsi}}$, updated at each iteration until convergence [43]. The social maternal effect was excluded for body width because the model did not converge, and for body length because it was not significant $\left( {\chi_{1DF}^{2} = 2.66, p = 0.264} \right).$ The fixed effects included for trait level and the variance part of the model were interaction of batch (2009, 2010, and 2011), sex (male and female), pond (1 and 2) and the linear covariate ‘age at harvest’.

To facilitate interpretation in the Results section, the group effect for trait level is presented as ${\text{g}}^{2} = {\hat\upsigma }_{\text{g}}^{2} /{\hat\upsigma }_{\text{P}}^{2}$, where $\upsigma _{\text{P}}^{2}$ is the phenotypic variance, and the kin effect as ${\text{k}}^{2} = {\hat\upsigma }_{\text{k}}^{2} /{\hat\upsigma }_{\text{P}}^{2}$. Moreover, for the genetic estimates, the genetic coefficient of variation (${\text{GCV}}$) for trait level and its residual variance (${\text{GCV}}_{\text{Ve}}$) are provided. These are defined as, ${\text{GCV}} = \upsigma _{\text{A }} /\mu$, where $\upsigma _{\text{A}}$ is the genetic standard deviation in trait level while $\mu$ is the population mean level of the trait [45], and, ${\text{GCV}}_{\text{Ve}} = \upsigma_{{{\text{A}}_{\text{V}} }} /\upsigma_{\text{E}}^{2}$, where $\upsigma _{{{\text{A}}_{\text{V}} }}$ is the genetic standard deviation in the residual variance and $\upsigma _{\text{E}}^{2}$ is the mean residual variance from the additive model [37, 46]. When $\upsigma _{{{\text{A}}_{\text{V}} }}^{2}$ is on the exponential scale, as is the case for the residual variance in our analysis, ${\text{GCV}}_{\text{Ve}}$ is close to $\sqrt {\upsigma _{{{\text{A}}_{\text{V}} }}^{2} }$ [37, 46].

## Results

### Genetic parameters for trait levels

Estimated genetic parameters for levels of harvest weight, body length, depth, and width are in Table 2. The estimated heritability for individual harvest weight (estimated by using the average residual variance across all observations) was equal to 0.25 (0.04) and the same value was obtained with a univariate model assuming a homogeneous residual variance (results not shown). The log-likelihood ratio tests indicated that both group and kin effects were highly significant (p < 0.0001). The group effect explained 13 % of the phenotypic variance, which shows that individuals within the same group are more similar to each other than to members of other groups. The kin effect explained 10 % of the phenotypic variance, which indicates that individuals within the same family are more alike compared to individuals of the other family in the group, in addition to their genetic similarity. We tested the model for harvest weight when group and kin effects were not included and found that removing one or both effects created an upward bias in the estimated variances for both the level and variance of the trait (results not shown). The social maternal effect was significant (p < 0.001) but small and explained 2 % of the phenotypic variance.

**Table 2:** Genetic parameters for level of harvest weight, length, depth, and width

Parameter	Harvest weight	Length	Depth	Width
^a^ $\upsigma _{{{\text{A}}_{ } }}^{2}$	573.46 (115.80)	0.732 (0.136)	0.202 (0.037)	0.034 (0.007)
$\upsigma _{{{\text{e}}_{ } }}^{2}$	1426.3 (27.99)	1.443 (0.028)	0.365 (0.007)	0.067 (0.001)
$\upsigma _{{{\text{g}}_{ } }}^{2}$	300.26 (42.81)	0.354 (0.047)	0.104 (0.012)	0.037 (0.004)
$\upsigma _{{{\text{k}}_{ } }}^{2}$	240.29 (35.45)	0.235 (0.035)	0.047 (0.008)	0.013 (0.002)
$\upsigma _{{{\text{m}}_{ } }}^{2}$	43.64 (20.83)	–	0.013 (0.006)	–
$\upsigma _{{{\text{P}}_{ } }}^{2}$	2297.2 (70.78)	2.418 (0.081)	0.631 (0.022)	0.136 (0.005)
${\text{h}}^{2}$	0.25 (0.04)	0.30 (0.05)	0.32 (0.05)	0.25 (0.05)
^b^ ${\text{g}}^{2}$	0.13 (0.02)	0.15 (0.02)	0.16 (0.02)	0.27 (0.02)
^c^ ${\text{k}}^{2}$	0.10 (0.02)	0.10 (0.01)	0.08 (0.01)	0.10 (0.01)
^d^ ${\text{m}}^{2}$	0.02 (0.01)	–	0.02 (0.01)	–
^e^ ${\text{GCV}}$	0.14	0.05	0.06	0.06

Standard errors are indicated between brackets

^a^Additive genetic variance was calculated as four times the sire-dam variance

^b^Group effect, calculated as ${\text{g}}^{2} = \upsigma _{\text{g}}^{2} /\upsigma _{\text{P}}^{2}$

^c^Kin effect, calculated as ${\text{k}}^{2} = \upsigma _{\text{k}}^{2} /\upsigma _{\text{P}}^{2}$

^d^Social maternal effect, calculated as ${\text{m}}^{2} = \upsigma_{\text{m}}^{2} /\upsigma _{\text{P}}^{2}$

^e^Genetic coefficient of variation

Heritabilities of harvest weight and body width were similar (0.25 ± 0.05), while heritabilities of body length and body depth were a little higher (~0.30 ± 0.05). The group effect explained ~15 % of the phenotypic variance for length and depth, and 27 % for width. The kin effect explained ~10 % of the phenotypic variance for all three body size traits.

### Genetic parameters for the variance of traits

Estimated genetic parameters for the variance of harvest weight, body length, depth, and width are in Table 3. For all traits, the contribution of genetic effects to their variance was highly significant (p < 0.0001). Estimated ${\text{GCV}}_{\text{Ve}}$ for harvest weight was high and equal to 0.58, whereas for body size traits, ${\text{GCV}}_{\text{Ve}}$ were lower i.e. 0.39, 0.42, and 0.45 for length, depth and width, respectively. These estimates indicate that there is substantial genetic variation in the residual variance compared to the average value of the residual variance, for all analyzed traits.

**Table 3:** Genetic parameters for the variance of harvest weight, length, depth, and width

Parameter	Harvest weight	Length	Depth	Width
^a^ $\upsigma _{{{\text{A}}_{ } }}^{2}$	0.343 (0.068)	0.156 (0.041)	0.184 (0.042)	0.203 (0.048)
$\upsigma _{{{\text{e}}_{ } }}^{2}$	1.747 (0.034)	1.924 (0.038)	1.862 (0.036)	1.696 (0.033)
^b^ $\upsigma _{{{\text{g}}_{ } }}^{2}$	0.040 (0.021)	0.031 (0.021)	0.031 (0.018)	0.073 (0.020)
^c^ $\upsigma _{{{\text{k}}_{ } }}^{2}$	0.078 (0.027)	0.098 (0.029)	0.022 (0.023)	0.062 (0.023)
^d^ $\upsigma _{{{\text{m}}_{ } }}^{2}$	0.009 (0.009)	–	0.023 (0.011)	–
^e^ ${\text{GCV}}_{{{\text{V}}_{\text{e}} }}$	0.58	0.39	0.42	0.45

Standard errors are indicated between brackets

^a^Additive genetic variance was calculated as four times the sire-dam variance

^b^Group variance

^c^Kin variance

^d^Social maternal variance

^e^Genetic coefficient of variation at variance level

### Genetic correlations between level and variance of traits

Estimated genetic correlations between level and variance for harvest weight and body size traits are in Table 4. The genetic correlation between level and variance for harvest weight was high and positive (0.60 ± 0.09), which implies that selection for increased harvest weight will also yield more variation in the level of this trait. For body size traits, genetic correlations between level and variance were lower than for harvest weight, and were not significantly different from 0 for length and width, but moderate and positive for depth (0.37 ± 0.13).

**Table 4:** Genetic correlations between level and the variance for harvest weight, length, depth, and width

Harvest weight	Length	Depth	Width
0.60 (0.09)	0.11 (0.16)	0.37 (0.13)	0.20 (0.15)

Standard errors are indicated between brackets

## Discussion

In this study, we used a DHGLM to estimate genetic variation in uniformity of harvest weight and three body size traits, i.e. length, depth, and width. Our results showed substantial genetic variation in uniformity of all analyzed traits, with ${\text{GCV}}_{\text{Ve}}$ ranging from 39 to 58 %, while ${\text{GCV}}_{ }$ for trait levels ranged from 5 to 15 %. A strong genetic correlation of 0.60 was found between trait level and variance, which suggests that selection for increased body weight at harvest will also result in more variation in the level of this trait.

### Heritability of individual harvest weight and body size traits

Estimated heritability for individual harvest weight was moderate (0.25 ± 0.04), which is similar to results from previous studies on Nile tilapia [2, 47, 48]. To date, the GIFT strain has undergone 14 generations of selection for harvest weight. Our findings, together with the small average inbreeding coefficient of 3.1 % in the analyzed GIFT population, suggest that there is still a considerable amount of genetic diversity available for further selection, which is also in agreement with the positive genetic trend observed in the GIFT strain [3].

Heritabilities for individual body size traits were also moderate (0.25 to 0.32), which provide opportunities to improve body size traits in Nile tilapia. Body size traits could become traits of interest in future breeding programs since selection for heavier fish may lead to body shapes that deviate from the natural shape, the latter being favored by consumers [13, 49, 50].

### Genetic variance in uniformity of harvest weight

Variance components that are estimated using the exponential model, as in this study, are independent of the scale of the trait, and thus, are comparable across traits and species [24, 30]. We found a substantial additive genetic variance for uniformity of harvest weight (0.34 ± 0.07; Table 3), which is larger than that in a similar study on Atlantic salmon by Sonesson et al. [30], who reported an additive genetic variance in the residual variance of 0.17 on the exponential scale. Our estimates are also higher than those reported for livestock traits [23, 24, 37, 51, 52]. These findings suggest that the observed phenotypic variability of harvest weight in the GIFT strain has a substantial genetic component.

Regardless of the underlying model, comparison of additive genetic parameters for uniformity across different studies can also be done by using the genetic coefficient of variation for residual variance (${\text{GCV}}_{\text{Ve}}$) [37, 46]. ${\text{GCV}}_{\text{Ve}}$ describes the change in residual variance when a genetic standard deviation of 1 is achieved in response to selection, relative to the mean of the residual variance. In our study, ${\text{GCV}}_{\text{Ve}}$ for harvest weight was large i.e. 0.58. The proportional change in phenotypic variance can be calculated as ${\text{GCV}}_{\text{Ve}} \left( {\upsigma _{\text{E}}^{2} /\upsigma _{\text{P}}^{2} } \right)$, which in the case of harvest weight would be equal to 0.36. In the literature, ${\text{GCV}}_{\text{Ve}}$ for variability of traits in livestock and laboratory animals usually ranges from 0.2 to 0.6 [37]. For uniformity of body weight in rainbow trout, ${\text{GCV}}_{\text{Ve}}$ of 0.37 and ~0.2 were reported by Janhunen et al. [29] and Sae-Lim et al. [31], respectively, which are lower than the values found in our study. The estimated ${\text{GCV}}_{\text{Ve}}$ for harvest weight suggests that there is sufficient genetic variation to allow a substantial change in the residual variance of this trait compared to its average value within a single generation of selection, which would be much larger than that for harvest weight level (Table 2). However, it should be noted that the accuracy of selection for uniformity tends to be lower than for trait levels [19], and that expressions for response to selection on environmental variability do not depend on ${\text{GCV}}_{\text{Ve}}$ only [7, 17, 27, 46].

### Effect of data distribution

The estimated level and variance for harvest weight could be influenced by the non-normal distribution of harvest weight. In data on aquaculture species, skewness is not unusual [6, 53]. A skewed distribution can result from inter-individual competition and subsequent feeding hierarchy, with a few individuals dominating the rest of the group. However, in many statistical inferences, normality is assumed and this is especially important in the analysis of the genetic heterogeneity of environmental variance [54]. To test whether genetic variation in residual variance is merely an artifact of a non-normal distribution, we applied a Box-Cox transformation to harvest weight. The transformation resulted in a normally distributed trait, which was then analyzed with the DHGLM. Results of the analysis (see Additional file 2: Table S1) showed that this transformation had only a minor effect on the estimated genetic parameters for trait level, but decreased the variance of the residual variance. Similar results were found in other studies that analyzed transformed traits [30, 31, 54]. Although the additive genetic variance of uniformity decreased somewhat after the Box–Cox transformation, this difference was not significant (p = 0.22). Thus, our results indicate that there is genetic variation in uniformity of harvest weight, irrespective of the scale of measurement of the trait. Unlike harvest weight, body length, depth, and width were normally distributed.

### Genetic correlation between level and variance of harvest weight and body size traits

Our results imply that the observed variation in harvest weight in the GIFT strain could be reduced by selective breeding. However, selection for more uniform fish may result in a trade-off on improvement of harvest weight. The genetic correlation between level and variance of harvest weight is high and positive, 0.60 (Table 4), which means that single-trait selection for heavier fish will increase the variation in harvest weight among individuals. Similar correlations were obtained by Sae-Lim et al. [31]. Simultaneous improvement of harvest weight and its uniformity will therefore require index selection.

To maximize profit, not only uniformity of weight but also uniformity of size, may play an important role in fish farming, especially for markets where fish are sold as whole. The magnitudes of ${\text{GCV}}$ for uniformity of body size traits and harvest weight were similar but improvement of body size traits based on the estimated correlations (Table 4) is expected to have a limited effect on the level of these traits.

### Factors affecting magnitude of variability and genetic variance in variability

In our analyses we used a sire and dam model, which fits the additive genetic mid-parent mean, while the Mendelian sampling deviation is part of the residual. This can potentially inflate the size of the estimated genetic variation in residual variance in case of heterogeneous Mendelian sampling variation, which is then confounded with the genetic part of the residual variance [30]. A Mendelian sampling variance that is heterogeneous among families can result from differential inbreeding coefficients of parents, or from the presence of a major gene that is segregating in some families but not in others [20].

In aquaculture species, maternal common environmental effects can have an important role in explaining differences among families. These effects can be included in the estimation of genetic parameters as non-genetic effects that account for covariances between full-sibs due to a shared environment. In this study, maternal common environmental effects were excluded from the models because of convergence problems, which arose when those effects were included. The same issue was observed in other studies that used the same dataset and for which the results showed confounding of maternal common environmental effects and direct genetic effects [12, 34]. The main difficulty that occurs when disentangling the two effects is due to the mating of one male with two females. Moreover, in our experiment, mating was often partly unsuccessful and resulted in 1 × 1 mating instead. However, even a perfect 2 × 2 mating design results in limited power to separate genetic and maternal common environmental effects, at least at the variance level, as reported by Sonesson et al. [30]. Previous studies on a larger GIFT population for which 1 × 2 mating was more successful, detected significant maternal common environmental effects (0.34) for individual harvest weight [2]. Thus, our estimates of the genetic variance of uniformity may be inflated by the inability to fit maternal common environmental effects.

A recent study on birth weight of mice treated environmental variability as a maternal trait, and found a positive response to selection [55]. In an earlier study, the same authors found evidence that environmental variability of birth weight was more likely to be a maternal genetic trait than a trait due to direct genetic effects [4]. In the study by Rutten et al. [56], the variance of body weight due to common environmental effects, which include maternal genetic and non-additive genetic effects, decreased with age. Since in our study, traits were measured at harvest, maternal genetic or common environmental effects probably explain only a small proportion of the heterogeneity of residual variance.

In Table S2 (see Additional file 3: Table S2), we present estimates of the fixed effects included in the model. All fixed effects had a significant impact on the magnitude of the observed variability. The effect of sex was especially large with males showing ~1.3 times greater residual variance compared to females. This finding may be related to the competitive behavior expressed primarily by males. Mulder et al. [19] showed that the estimated genetic correlation between residual variances for body weight of both sexes was only 0.11, which suggests that they are different traits. A similar analysis could be conducted on our data, to investigate whether the large effect of sex is associated with a genetic correlation for variability between sexes that is less than 1. Ponzoni et al. [2] recorded the CV of body weight in the GIFT strain across eight generations and observed that good breeding management contributed to reduce the CV, although its average value remained at around 40 %. Thus, reducing uniformity will require both genetic and management interventions.

### CV for harvest weight

In our experiment, the feeding strategy differed from that in the ordinary GIFT breeding program. Instead of spreading food on the surface of the pond as in the GIFT breeding population, we placed it in the corner of the net-cages so that the fish showed their competitive tendency. The CV for harvest weight in our study (35 %) was lower than the values found in previous studies on the GIFT strain where fish were communally reared (48 to 59 %) [10, 50]. Thus, there is no evidence that the level of competition between individuals was higher in our conditions than in the communal rearing conditions of these studies. In communal rearing, the feed is not spread over the entire pond’s surface because auto-feeders are not available, which may cause some competition. In addition, the fish in our experiment were kept in small net-cages and stocked at low density, while in commercial ponds all fish are kept together at high density. Because of the differences in rearing conditions, the question of whether our results can be extended to commercial situations remains open. A selection experiment, in which parents are kept in many small groups and selected for uniformity while offspring are evaluated under commercial conditions, would constitute the ideal proof.

### Future prospects

Although studies on the genetic heterogeneity of environmental variance date as far back as 1942 [57], selection experiments to improve uniformity in livestock are scarce. Still, some experiments [58–61] that were based on divergent selection for phenotypic variance, provided evidence for a genetic component in the phenotypic variability and suggested the possibility that this variability could be reduced by selective breeding. To our knowledge, selection for uniformity has never been performed in aquaculture species. Nevertheless, the high ${\text{GCV}}_{\text{Ve}}$ found in our and other studies on aquaculture species suggest that aquaculture populations are suitable to validate the estimated genetic parameters by a selection experiment. Selection for uniformity of body weight or size could lead to increased profit by producing more fish in the size range that is favored by the consumers. Moreover, from the point of view of animal welfare, uniformity of fish body weight and size could reduce competition, and thus possible stress, injuries, and even mortality.

We studied the genetic variance of the residual variability. However, the total phenotypic variability also depends on other factors [62], as shown by the significant fixed effects on variability, for example sex effect (see above). Hence, decreasing the total phenotypic variability even more would require reducing the magnitude of these fixed effects. When the genetic correlation between growth rate in males and females differs from 1, it is possible, in principle, to remove the variability due to a difference in mean body weight between sexes. The magnitude of environmental effects, such as group and batch effects, is related to environmental sensitivity (and thus to genotype by environment interactions). Evaluating the prospects of reducing these components by genetic selection will require further research.

An interesting property of the specific design of our experiment is that it allows the simultaneous study of uniformity and social effects such as group and kin effects in our study and indirect genetic effects, which were analyzed in other studies on the same data [12, 34]. However, the experimental setting and feeding strategy that we applied differed from those in a commercial setting. Thus, genotype by environment interactions may be present and our results may not represent uniformity in the case of commercial tilapia farms. The DHGLM approach could be used to test whether the genetic background of uniformity differs between both environments. Results from such a study would be a useful addition to our findings.

## Conclusions

Our study revealed substantial genetic variation in uniformity of harvest weight and body size traits, which opens promising prospects for the genetic improvement of uniformity by selective breeding of the GIFT strain. The genetic correlation between level and variance of harvest weight was high and positive, which indicates that selection for heavier fish may also result in more variation in harvest weight. Simultaneous improvement of harvest weight and its uniformity will thus require index selection.

## Additional files

10.1186/s12711-016-0218-9 Example of the block design used to allocate two families to each group. Figure S1 represents an example of block design used to allocate two families to each group. All families in the block are unrelated to each other.
10.1186/s12711-016-0218-9 Genetic parameters for the mean and the variance of Box-Cox transformed harvest weight. Table S1 contains genetic parameters for the mean and the variance of Box-Cox transformed harvest weight estimated using a double hierarchical generalized linear model.
10.1186/s12711-016-0218-9 Estimates of fixed effects for variance of the level of harvest weight, length, depth, and width. Table S2 contains estimates of the fixed effects for variance of the level of harvest weight, length, depth, and width, obtained from the reduced model with main fixed effects (not the interactions) and random effects.
