# Supplementary material for: Genetic parameters for uniformity of harvest weight and body size traits in the GIFT strain of Nile tilapia
Source: Genet Sel Evol. 2016 Jun 10;48:41. doi: 10.1186/s12711-016-0218-9 (PMC4901462; doi:10.1186/s12711-016-0218-9)
Supplement: Supplementary file 1 — 10.1186/s12711-016-0218-9 Example of the block design used to allocate two families to each group. Figure S1 represents an example of block design used to allocate two families to each group. All families in the block are unrelated to each other. [file 12711_2016_218_MOESM1_ESM.pdf]

Additional file 1

Figure S1 represents example of block design used to allocate two families to each group. All families in the block are unrelated to each other.

**Family ID**	**2**	**3**	**4**	**5**	**6**	**7**	**8**	**9**	**10**	**11**
**1**	1-2	1-3	1-4	1-5	1-6	1-7	1-8	1-9	1-10	1-11
**2**		2-3	2-4	2-5	2-6	2-7	2-8	2-9	2-10	2-11
**3**			3-4	3-5	3-6	3-7	3-8	3-9	3-10	3-11
**4**				4-5	4-6	4-7	4-8	4-9	4-10	4-11
**5**					5-6	5-7	5-8	5-9	5-10	5-11
**6**						6-7	6-8	6-9	6-10	6-11
**7**							7-8	7-9	7-10	7-11
**8**								8-9	8-10	8-11
**9**									9-10	9-11
**10**										10-11

Figure S1 Example of the block design used to allocate two families to each group
