# Supplementary material for: Genetic parameters for uniformity of harvest weight and body size traits in the GIFT strain of Nile tilapia
Source: Genet Sel Evol. 2016 Jun 10;48:41. doi: 10.1186/s12711-016-0218-9 (PMC4901462; doi:10.1186/s12711-016-0218-9)
Supplement: Supplementary file 2 — 10.1186/s12711-016-0218-9 Genetic parameters for the mean and the variance of Box-Cox transformed harvest weight. Table S1 contains genetic parameters for the mean and the variance of Box-Cox transformed harvest weight estimated using a double hierarchical generalized linear model. [file 12711_2016_218_MOESM2_ESM.pdf]

Additional file 2

Table S1 contains genetic parameters for the mean and the variance of Box-Cox transformed harvest weight estimated using Double Hierarchical Generalized Linear Models.

Table S1 Genetic parameters for the mean and the variance of Box-Cox transformed harvest weight

Parameter	Mean	Variance
[a]σ_A^2	0.100 (0.019) [b]	0.239 (0.052)
σ_e^2	0.184 (0.004)	1.804 (0.035)
σ_g^2	0.050 (0.007)	0.035 (0.019)
σ_k^2	0.032 (0.004)	0.048 (0.025)
σ_m^2	0.008 (0.003)	0.014 (0.009)
σ_P^2	0.323 (0.011)	_
h^2	0.31 (0.05)	_
[b]g^2	0.15 (0.02)	_
[c]k^2	0.10 (0.02)	_
[d]m^2	0.02 (0.01)	_
[e]GCV	0.06	0.49

Standard errors are indicated between brackets
[a]Additive genetic variance was calculated as 4 times the sire-dam variance
[b]Group effect, calculated as $g^2 = \sigma_g^2/\sigma_P^2$
[c] Kin effect, calculated as $k^2 = \sigma_k^2/\sigma_P^2$
[d] Social maternal effect, calculated as $m^2 = \sigma_m^2/\sigma_P^2$
[e] Genetic coefficient of variation
