# Supplementary material for: Genetic parameters for uniformity of harvest weight and body size traits in the GIFT strain of Nile tilapia
Source: Genet Sel Evol. 2016 Jun 10;48:41. doi: 10.1186/s12711-016-0218-9 (PMC4901462; doi:10.1186/s12711-016-0218-9)
Supplement: Supplementary file 3 — 10.1186/s12711-016-0218-9 Estimates of fixed effects for variance of the level of harvest weight, length, depth, and width. Table S2 contains estimates of the fixed effects for variance of the level of harvest weight, length, depth, and width, obtained from the reduced model with main fixed effects (not the interactions) and random effects. [file 12711_2016_218_MOESM3_ESM.pdf]

# Additional file 3

Table S2 contains estimates of the fixed effects for variance level of harvest weight, length, depth, and width, obtained from the reduced model with main fixed effects (not the interactions) and random effects

**Table S2** Estimates of fixed effects for variance level of harvest weight, length, depth, and width

| Effect | | Variance level of HW | | | Variance level of length | | | Variance level of depth | | | Variance level of width | | |
|---|---|---|---|---|---|---|---|---|---|---|---|---|---|
| | | Estimate | SE | Fraction[a] | Estimate | SE | Fraction | Estimate | SE | Fraction | Estimate | SE | Fraction |
| Sex | F | 0.0 | 0.0 | 1.0 | 0.0 | 0.0 | 1.0 | 0.0 | 0.0 | 1.0 | 0.0 | 0.0 | 1.0 |
| | M | 0.558 | 0.036 | 1.747 | 0.224 | 0.078 | 1.252 | 0.229 | 0.036 | 1.257 | 0.181 | 0.035 | 1.198 |
| Batch | 2009 | -0.605 | 0.231 | 0.546 | -0.033 | 0.192 | 0.968 | -0.318 | 0.191 | 0.728 | -0.353 | 0.195 | 0.703 |
| | 2010 | 0.0 | 0.0 | 1.0 | 0.0 | 0.0 | 1.0 | 0.0 | 0.0 | 1.0 | 0.0 | 0.0 | 1.0 |
| | 2011 | 0.255 | 0.115 | 1.290 | 0.081 | 0.103 | 1.084 | 0.017 | 0.103 | 1.017 | 0.095 | 0.104 | 1.099 |
| Pond | 1 | 0.0 | 0.0 | 1.0 | 0.0 | 0.0 | 1.0 | 0.0 | 0.0 | 1.0 | 0.0 | 0.0 | 1.0 |
| | 2 | -0.376 | 0.049 | 0.687 | -0.163 | 0.047 | 0.791 | -0.112 | 0.045 | 0.894 | -0.167 | 0.048 | 0.846 |
| age | | 0.008 | 0.001 | 1.008 | 0.001 | 0.001 | 1.001 | 0.003 | 0.001 | 1.003 | 0.004 | 0.001 | 1.004 |

[a] Exponent of the estimate
